# Dysregulation of inflammasome activation in glioma

**DOI:** 10.1186/s12964-023-01255-5

**Published:** 2023-09-18

**Authors:** JeongMin Sim, JeongMan Park, Jong-Seok Moon, Jaejoon Lim

**Affiliations:** 1 Department of Biomedical Science, College of Life Science, CHA University, Pocheon, 11160 Republic of Korea; 2 Department of Neurosurgery, CHA Bundang Medical Center, CHA University College of Medicine, 59 Yatap-Ro, Bundang-Gu, Seongnam, 13496 Republic of Korea; 3 Department of Integrated Biomedical Science, Soonchunhyang Institute of Medi-Bio Science (SIMS), Soonchunhyang University, Cheonan, 31151 Republic of Korea

**Keywords:** Glioma, Neuroinflammation, Inflammasome, Therapeutic strategy

## Abstract

Gliomas are the most common brain tumors characterized by complicated heterogeneity. The genetic, molecular, and histological pathology of gliomas is characterized by high neuro-inflammation. The inflammatory microenvironment in the central nervous system (CNS) has been closely linked with inflammasomes that control the inflammatory response and coordinate innate host defenses. Dysregulation of the inflammasome causes an abnormal inflammatory response, leading to carcinogenesis in glioma. Because of the clinical importance of the various physiological properties of the inflammasome in glioma, the inflammasome has been suggested as a promising treatment target for glioma management. Here, we summarize the current knowledge on the contribution of the inflammasomes in glioma and therapeutic insights.

Video Abstract

**Supplementary Information:**

The online version contains supplementary material available at 10.1186/s12964-023-01255-5.

## Introduction

Glioma is the most prevalent intracranial brain tumor (comprising 81% of malignant brain tumors) that is thought to be driven by neuroglial or progenitor cells [1]. Although optimal standard treatments based on the biological or clinical background of glioma have been developed, the prognosis has not drastically improved due to the complicated heterogeneity and aggressive microenvironment of the glioma [2]. Stagnant survival statistics and increased malignancy demonstrate the urgent need for continued research to develop more effective therapies for glioma [1].

The tumor microenvironment (TME) created by interactions between malignant and non-transformed cells acts as a host supporting the expansion and invasion of tumors, promoting neoplastic transformation, protecting the tumor from host immunity, and providing niches for dormant metastases to flourish [3, 4]. Among the highly heterogeneous elements of the TME, an aggressive inflammatory process is one of the vital elements leading to dismal treatment results of glioma and glioblastoma [5]. Malignant progression of glioma relates to a neuroinflammatory response, deemed as a hallmark of tumor growth, invasion, angiogenesis, and metastasis [6]. A neuroinflammation-enriched TME developed through the production of pro-inflammatory cytokines, chemokines, and growth factors facilitates an immune-suppressive response and aids in the survival capacity of glioma cells [7, 8]. The clinical significance of treatment of chronic inflammation in the central nervous system (CNS) shows therapeutic potential for gliomas at the molecular level [9].

The association between neuroinflammation and the inflammasome (multiprotein complex) involved in the innate immune system is an emerging research topic in glioma [10, 11]. Mounting evidence for an inflammasome-mediated inflammatory response describes the erroneous functions of the innate immune system in glioma TME [12]. Generation of proinflammatory cytokine by inflammasome activation can promote glioma progression [12]. Because the inflammasome has clear biological implications in glioma, clinical studies on its identity are essential. In this review, we discuss the latest insights into the function and molecular mechanism of inflammasomes in glioma, suggesting a therapeutic approach on a molecular level.

### Pathology of glioma

The common symptoms of glioma include seizure, cognitive disorder, aphasia, motor paresis, and headache [13]. Physical diagnosis of patients with glioma, including those with glioblastoma, the most aggressive form of glioma, is based on detecting the pathological origin and specific subtype of cancer via neurosurgical procedures and molecular and histological examinations [14]. Based on the diagnosis using computed tomography or magnetic resonance imaging assessments, treatment decisions involving surgical resection, radiotherapy, and TMZ chemotherapy are suitably established [14]. In 2021, the fifth edition of the World Health Organization (WHO) classification of CNS was published, which contains general changes, including the taxonomy and nomenclature of glioma [15]. The novel stratification of glioma with 1p/19q co-deletion based on fluorescence in situ hybridization (FISH) analysis and IDH mutant or wildtype based on IHC analysis became more sophisticated with additional diagnostic evaluation indices such as loss of ATRX expression or *TERT* promoter mutations, the presence of *TP53* or histone H3 mutations, *EGFR* amplification, and *CDKN2A/B* alterations [16]. Based on the newly developed criteria, numerous biological classifications and treatment strategies are being proposed [15, 16]. These advances have resulted in an improved understanding of the molecular pathogenesis of glioma with somatic mutations, hyperinflammatory responses, metabolic dysfunction, immunoediting, and cell plasticity.

Large-scale efforts have been made to identify the major genetic and epigenetic alterations in glioma [17]. The data from The Cancer Genome Atlas (TCGA) and Chinese Glioma Genome Atlas (CGGA) project has aided in understanding the molecular landscape of glioma, allowing the establishment of several subtypes and genomic characteristics [18, 19]. In line with previous reports, the status in *IDH1/2*, *1p/19q*, *TP53*, *CIC*, *PTEN*, *EGFR*, *MGMT*, *TERT*, *ATRX*, and *Ras/MAPK* and extrachromosomal DNA were used as pathological indications of glioma [20–22]. These approaches further subdivided the glioma molecular subtypes into neural, proneural, classical, and mesenchymal types [15, 23].

Until recently, histological examination was one of the “gold standards” for diagnosing glioma [24]. Gliomas are generally graded using WHO grades 1–4 based on malignancy signatures, including the degree of mitotic activity, atypia, microvascular proliferation, pseudopalisading necrosis, and specific hallmarks [15]. Although this histological classification has developed over the years, it has some limitations, such as interobserver variability and apprehensive subject quality during in vitro examinations [25]. Thus, to improve our understanding of histological information, molecular features and clinical opinions should be considered together [26].

For several decades, the cellular origin of glioma has been a hot topic of interest in tumorigenesis in the CNS [27]. Numerous scholars postulate that gliomas originate from neural stem cell (NSC) lineages such as neurons, oligodendrocyte precursor cells (OPCs), oligodendrocytes, and astrocytes [27, 28]. OPCs expressing NG2, OLIG2, A2B5, and PDGFRα are the most abundant cells in CNS, and the proliferation of adult OPCs may play a pathological role in glioma development via responses to bFGF and PDGF-AA [29–31]. Astrocytes were identified as the causative cells of gliomas in the 1980s [32], and features such as mutated epidermal growth factor receptor (EGFR) and activation of H-RAS, considered representative signatures of gliomas, were revealed in a mouse model [33–35]. It has been verified in animal models that other cells, such as glial restricted progenitor cell (GRPC) and astrocyte precursor cell (APC), are cells of glioma origin [36].

The TME throws the physiological phenotype into disorder by closely interacting with diverse elements [37]. This is no different in glioma; in fact, the TME in glioma has a complex heterogeneity that is difficult to understand [38, 39]. Large-scale studies conducted to understand the TME and reduce interindividual variability have provided a fragmented genetic status of cells [40, 41]. Aggressive genetic and phenotypic profiles of glioma with proliferative, invasive, and immune-suppressive signatures contribute to the formation of a malignant signaling axis via the acceleration of an autocrine or paracrine loop [42–44]. These common features of gliomas represent a fundamental baseline for standard treatment and follow-up management [45, 46]. In recent years, single-cell RNA sequencing (scRNA-seq) has allowed the study of the biological properties of individuals with unprecedented resolution [47–49]. Single-cell landscapes, supplemented with bulk RNA-seq and histological staining results, have provided insights into the TME, including details regarding the functions of specific cells and molecules [47, 50]. To date, the progress has closely revealed pathological characteristics of specific molecular subtypes, cell types, and molecules within gliomas [15, 51]. In particular, the mesenchymal signature of glioma (also called mesenchymal subtype) exhibits a high inflammatory response, potential cellular plasticity, BBB instability, and immune infiltration, and myeloid lineage cells, including microglia and macrophages, may play a role in increasing the malignancy of the tumor microenvironment [52–54]. In addition, positionally resolved multi-omics with spatial transcriptomic analysis may help decipher the tumoral development process and bidirectional cell-to-cell interdependence in the TME of gliomas [55, 56]. Dissecting the composition and functional heterogeneity of the TME of tumor cells and infiltrating cells would extend our understanding of glioma and allow us to improve the therapeutic efficacy for good prognosis of patients.

### Neuroinflammation in glioma

Classically, the CNS was supposed to be an “immune privileged” site following the rejection of transferred foreign tissue into the brain [57]. This is due to a specialized microenvironment including the BBB, inner blood-retinal barrier, low MHC expression, draining lymphatic insufficiency, specialized antigen-presenting cells, and plentiful anti-inflammatory modulators to protect normal neurons from aggressive immune responses [58, 59]. Although the characteristics of the natural status of the CNS are necessary for the maintenance of the environmental composition and proper function, these can be fatal in CNS diseases [60]. Importantly, studies for CNS disease, including glioma have focused on the pathogenic alteration of inflammatory response in the TME [61].

Neuroinflammation is closely linked to the vascular barrier. BBB is a selectively permeable barrier secured by endothelial cells linked to each other by tight junctions, a pericyte-embedded layer, and an astrocyte end-feet anchor [60, 62]. In the glioma, endogenous or exogenous pathogenic stimuli cause devastating neuroinflammation, altering the BBB cell layer characteristics and permeability of blood vessels [10, 63, 64]. A compromised BBB by an enhanced inflammatory response is influenced by diverse elements, including TNF-α, IL-1β, TGF-β, HIF-1α, VEGF, and metalloproteinase induced by inflamed immune cells [65–67]. Although BBB breakdown by neuroinflammation supports the progression of glioma in an autocrine and paracrine manner, with respect to immunotherapy, it is paradoxically that it allows for easy infiltration of peripheral immune cells to the CNS [68, 69].

Inflammatory response mediators form an important checkpoint in glioma. At the cellular level, myeloid cells (~ 60% of immune cells in glioma) are the most common immune cell type in glioma [10]. Representatively, proliferative and pro-inflammatory microglial cells (called brain resident myeloid cells) have a significantly positive correlation with glioblastoma progression [70]. Interestingly, microglia and macrophages can be polarized into two different phenotypes (M1: pro-inflammatory role, M2 type: anti-inflammatory role), and the M1/M2 ratio significantly affects the neuroinflammatory microenvironment [71]. These cells release inflammatory cytokines and chemokines such as IL-1β, TNF-α, IL-6, IL-12, IL-23, CCL2, CCL3, CCL4, CCL5, CXCL10, and CCL12, causing neuroinflammatory disorders [72]. Furthermore, glioma-associated microglia/macrophages (GAMs), which account for approximately 30% of the surgically resected glioma mass, play a key role in neuroinflammation [10]. GAMs not only produce immunosuppressive cytokines and tumor growth factors favorable for tumor growth but promote glioma progression by indiscriminate induction of inflammatory cytokines [73, 74]. In this regard, therapeutic strategies targeting specific molecules expressed primarily in these cells have been described considerably [75]. In contrast, a chronic inflammatory response in glioma and glioblastoma promotes the accumulation and activation of MDSCs, which inhibits anti-tumor immunity [76]. These cells are recruited by SDF-1, CCL2, and CXCL2 and then proliferate in response to IL-6, VEGF, GM-CSF, and PGE2 released by the glioma cells, further compromising the inflammatory microenvironment [77–79]. Astrocytes, known to be the most closely related to neuroinflammation among cells not of the myeloid lineage, originally orchestrate neuronal development by secreting synaptogenic molecules and pruning excess synapses [80–82]. Among diverse astrocyte populations, reactive astrocytes (upregulated GFAP astrocyte) exhibit neurotoxic activity in various neurodegenerative diseases and promote inflammatory signaling pathways, including JAK/STAT3, calcineurin, NF-κB, and MAPK pathways [83, 84]. Taken together, the pathological processes of these cells, mediating an inflammatory response or induced by inflammation, may enhance the proliferation, invasion, chemoresistance, and immune protection of tumor cells in glioma.

**Fig. 1:**
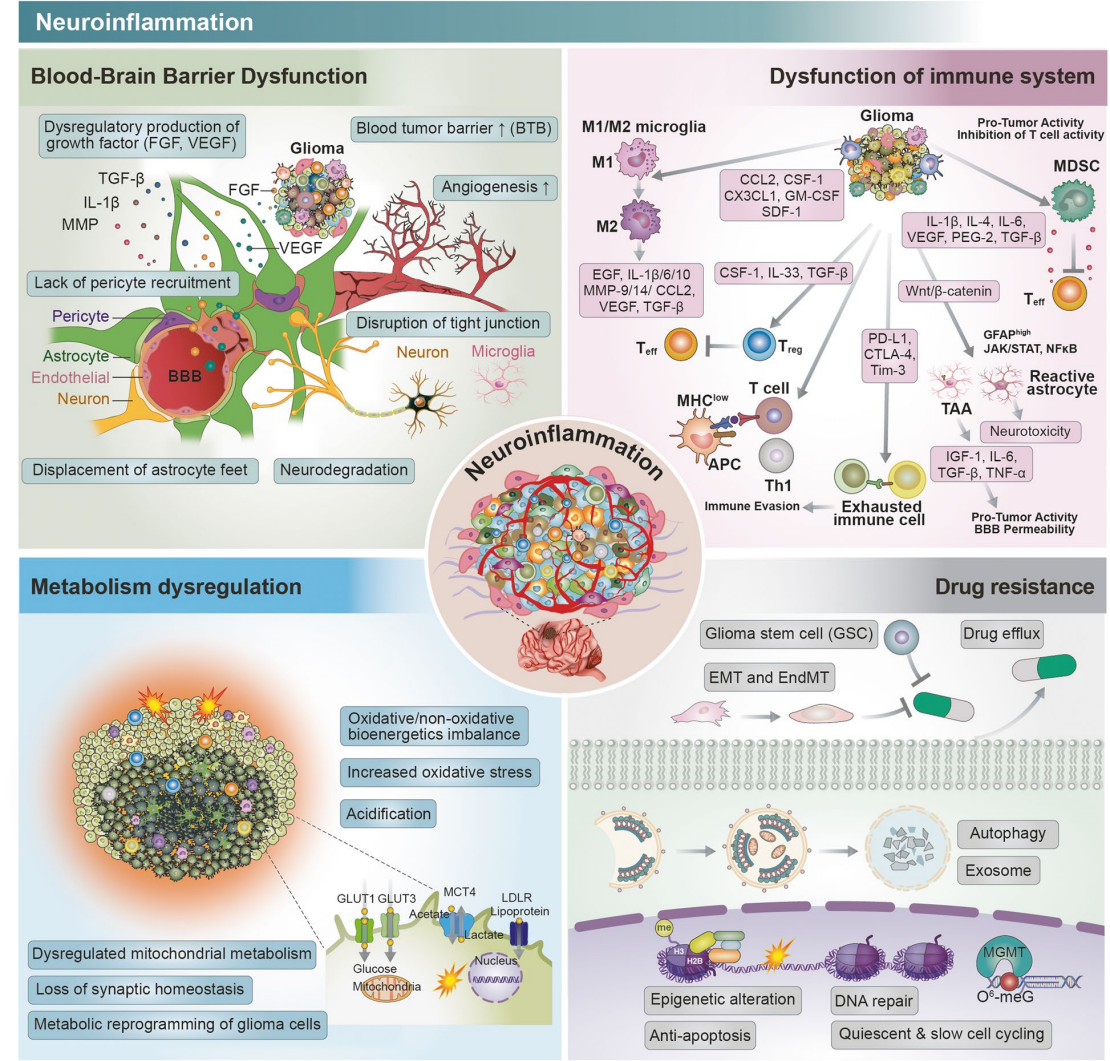
Devastating roles of neuroinflammation in glioma. The inflammatory response in glioma can induce BBB dysregulation (green section). Aggressive neuroinflammation of the glioma immune microenvironment exhibits intricate heterogeneity (purple section). In view of metabolism dysregulation, neuroinflammation convert metabolic balance of glioma cells (blue section). Glioma with chronic inflammation exhibits significant resistance to chemo/immuno-therapy (gray section)

To completely understand the process of neuroinflammation in glioma, the molecular network must be approached as a means of understanding cell-to-cell communication [85]. The functional and phenotypic landscapes of the cytokines, chemokines, growth factors, cis or trans elements, and cytosolic nucleic acids involved in the inflammatory microenvironment have already been specifically described for CNS diseases [85]. These molecules are not limited to the inflammatory response but show associations with various mechanisms such as metabolism, homeostasis, DNA repair, cell plasticity, and immunomodulation and form intra- or inter-links with these [86]. Therefore, discovery and validation of novel mechanisms in glioma, as well as identification of collaboration between them, will provide the foundation for a complete understanding of the inflammatory microenvironment in gliomas (Fig. 1).

### Association of the inflammasome with glioma

**Fig. 2:**
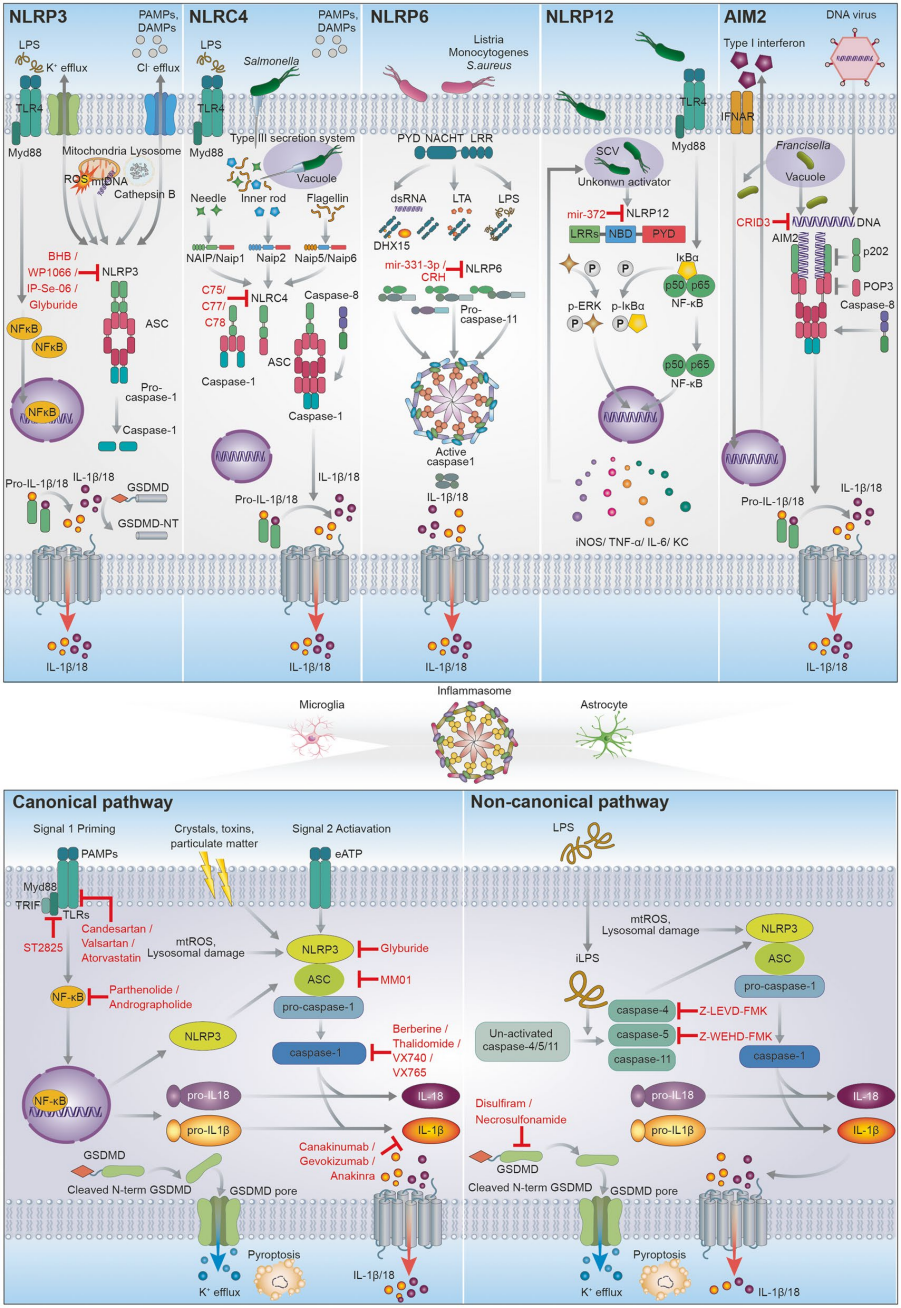
Schematic representation showing that general structure, assembly and activating pathway (canonical and non-canonical) for inflammasome in glioma. Potential therapeutic candidate agents for inflammasome targets were suggested in red marker

Inflammasomes are intracellular multimeric protein complexes comprising a NOD-like receptor (NLR), adaptor apoptosis-associated speck-like protein (ASC), and pro-caspase-1, which were discovered in 2002 [87]. They elicit the innate immune response via caspase-1 cleavage and secretion of pro-inflammatory cytokines such as IL-1β and IL-18 against pathogenic microorganisms or danger signals [88]. Based on the general background of the inflammasome in various diseases, reports of associations with glioma have been analyzed, and we have discussed the advances in the overall view of the inflammasome in glioma (Fig. 2).

As with other tumors or autoimmune diseases, various inflammasomes, including NLRP3 and NLRC4, have been reported in glioma. Although attention has been paid to the differences in the composition and function of inflammasomes due to the spatial specificity of the CNS bearing the BBB, the inflammasomes are highly conserved across tissues and cell types [12]. Assembly and activation of the inflammasome is a key function mediated by the innate immune response, and recent advances have significantly contributed to understanding the macromolecular identity of the inflammasome in glioma [12]. Representative upstream signals that induce the inflammasome are known to be pattern recognition receptors (PRRs) such as the Toll-like receptor (TLR) and RIG-I-like receptor (RLR) families [89, 90]. Current reports have suggested the contribution of the PRRs acting in a paracrine fashion to the development and malignancy of glioma [91–94]. In particular, the TLR families, including TLR1, TLR2, TLR3, TLR4, TLR6, and TLR9, are highly expressed in the TME of glioma, both in vitro and in vivo, leading to neuroinflammation [93, 95–100]. Mounting expression of TLRs in numerous cell types of glioma TME such as microglia, plasmacytoid DCs (pDCs), glioma stem cell (GSCs), GAMs, and astrocytes accelerates the network of signaling events that result in de novo synthesis via transcriptional modulation or non-proteinaceous signaling molecules [96, 101–107]. Upon PRR sensing of certain stimuli, the NF-κB signaling pathway is activated, which further induces NLR protein (known as inflammasome receptor protein) transcription, pro-IL-1β and pro-IL-18 transcription, and the inflammatory response both locally and systemically through a positive feedback loop [101]. Importantly, these steps are defined as the priming step (also called the first step of inflammasome assembly and activation), which allows the maintenance of constitutively high levels of pro-inflammatory cytokines and inflammasome sub-molecules. The NF-κB pathway, a key element of the priming step of the inflammasome, plays a pathological role in gliomas [108]. Meanwhile, the recognition of PAMPs or danger signals by a unique PRR results in proper assembly and activation of inflammasomes (second step of inflammasome activation) [109]. Although there are fundamental differences between inflammasomes that depend on stimuli, generally, canonical inflammasomes serve as a scaffold to recruit adaptor proteins known as ASCs, which consist of two death-fold domains, a pyrin domain (PYD) and a caspase recruitment domain (CARD), and inactive zymogen pro-caspase-1 [110]. Subsequent oligomerization of pro-caspase-1 induces their autoproteolytic cleavage into active caspase-1 [111]. Activated caspase-1 is a cysteine-dependent protease that cleaves the precursor cytokines pro-IL-1β and pro-IL-18, generating the active forms of IL-1β and IL-18, respectively [111]. At present, the intermediate and final products formed during inflammasome assembly and activation have been identified as important growth- and motility-driving elements in gliomas [112, 113]. Although the mechanisms underlying the glioma inflammasome activation remain unclear, the recruitment and activation of the component molecules of the inflammasomes are associated with malignancy in gliomas [12]. For example, berberine treatment inhibits glioma growth by inactivating caspase-1-mediated inflammatory cytokines via ERK1/2 regulation [114]. In this regard, the pharmacological inhibition of the inflammasome receptor protein, adaptor protein, caspase-1, and pro-inflammatory cytokines may facilitate glioma management.

To date, numerous receptor proteins of inflammasome assembly have been identified, including NLRP1, NLRP2, NLRP3, NLRC4, NLRP6, NLRP12, AIM2, IFI16, and pyrin [110]. As described above, these proteins recruit adapter proteins and inactive caspase-1 to assemble an inflammasome platform. Importantly, two pathways called canonical and non-canonical pathways are involved in the subsequent inflammasome activation process [111]. Recent developments toward our understanding of the canonical pathway (the inflammasome-caspase-1-proinflammatory cytokine axis) of inflammasome activation in glioma have been expertly reviewed in depth [111]. However, the role of the non-canonical inflammasome pathway in glioma is still unclear. In general, the non-canonical inflammasome pathway, which targets caspase-11 (in mice), caspase-4 (in human) and caspase-5 (in human), can restore the activation of the canonical inflammasome pathway [115]. Direct sensing of LPS and gram-negative bacteria by caspase-4 or caspase-5 induces cleavage and oligomerization of caspase-4 or caspase-5 [115]. These active forms directly promote pyroptosis, called pro-inflammatory cell death, via cleavage of the pore-forming protein gasdermin D (GSDMD) [115]. The non-canonical inflammasome pathway, similar to the canonical inflammasome pathway, is intertwined with pathological functions in CNS diseases [116, 117]. However, the overall mechanism of action of the non-canonical inflammasomes in glioma has not been presented, but the functions of the individual molecules associated with them have been investigated. In fact, 15 differentially expressed genes, including caspase-5, were upregulated in glioma tissues (n = 667) compared with those in normal brain tissues (n = 1152), suggesting a prognostic value for the pyroptosis-related gene signature in glioma [118]. Cox regression, Kaplan–Meier analysis, and IHC results showed that GSDMD might be a novel biomarker for the prognosis and TMZ sensitivity in glioma [119]. There is additional evidence from a computational analysis that GSDMD is significantly positively correlated with glioma malignancies [120].

### Role of inflammasomes in glioma

#### NLRP3 inflammasome

Among the inflammasomes, the NLRP3 inflammasome is the most analyzed protein complex [121]. Generally, NLRP3 is induced by a signal through stimulation of TLRs, NLRs, and cytokine receptors in the myeloid cell lineage, which undergo a priming step (which is an initiation activation) and are subsequently activated by NLRP3 stimulators, including extracellular pathogens, ATP, RNA–DNA hybrids, ionic flux, mitochondrial dysfunction, reactive oxygen species (ROS), and lysosomal damage, to mediate the innate immune response [121]. Since 2014, the function of the NLRP3 inflammasome in gliomas has been closely investigated. Aggressive expression and activity of the NLRP3 inflammasome were observed in cells derived from glioma patients [113], suggesting that the NLRP3 inflammasome is a potential marker of glioma progression [113, 122]. Ever since the pathological function of NLRP3 in glioma was understood, its regulatory mechanisms and potential as a therapeutic target have been evaluated. Emerging evidence regarding the regulatory mechanism of the NLRP3 inflammasome has shown that NLRP3 can induce the EMT and PTEN/AKT signaling pathways and lead to glioma cell proliferation, apoptosis, and metastasis [123]. Alendronate (ALD: one of the nitrogen-containing bisphosphonates) treatment of glioma cell line causes augmented NLRP3 inflammasome activity, apoptosis, and mitochondrial damage, indicating that ALD is associated with impairment of the mevalonate pathway, which inhibits cholesterol synthesis and protein prenylation [124]. Another report has exhibited that the NLRP3 inflammasome induces proliferation and invasion of glioma cells via regulation of IL-1β and NF-κB p65 signaling [125]. Importantly, a report on the upstream signaling pathway of the NLRP3 inflammasome in gliomas has been published, and activation of the ERK-dependent NF-κB has been shown to activate the NLRP3 inflammasome mediated by vimentin in EV-71-infected glioma [126]. Considering these findings, the outline of the potential axis of the NLRP3 inflammasome involved in the induction and regulation of gliomas has been revealed.

In addition to the previous work, studies to build basic knowledge on NLRP3 inflammasome-targeted therapeutic approaches in gliomas have been performed in parallel. Beta-hydroxybutyrate (BHB) inhibits the migration of glioma by suppressing NLRP3 inflammasome expression and activation [127]. WP1066, which inhibits the activation of STAT3 by directly targeting JAKs, also suppresses glioma cells via NLRP3 inflammasome inhibition independently of STAT3 inhibition [128]. Additional evidence suggests that NLRP3 inflammasome blockade therapy using IP-Se-06 (selenylated imidazo[1,2-*a*]pyridine) induces anti-proliferation of glioma cells via inhibition of p38 MAPK and p-p38, leading to inhibition of the NLRP3 inflammasome [129]. In addition, in-depth glioma studies were conducted on the effects and mechanisms of the NLRP3 inflammasome in terms of cellular plasticity including M1 macrophage polarization and drug resistance [130, 131]. The simultaneous physiological, etiological, and therapeutic approaches targeting the NLRP3 inflammasome in glioma have led to remarkable progress, and efforts are being made to fully understand the role of the NLRP3 inflammasome through a translational study based on previous studies.

#### NLRC4 inflammasome

The origins of NLRC4 inflammasome have been explored earlier along with that of NLRP3. Various approaches to investigate its structure and function have been attempted [132–135]. In general, the NLRC4 inflammasome is involved in innate immunity against pathogens such as bacterial flagellin and T3SS needle and rod protein [135]. While it is closely modulated by transcriptional regulation, post-translational modification (PTM), specific phosphorylation, and ubiquitination, it promotes the pathogenesis of various autoimmune diseases and tumors due to its abnormally high expression and dysregulation [136–138].

The role of the NLRC4 inflammasome in gliomas was first described in 2019, and our findings identified that robust expression and activation of NLRC4 are associated with glioma progression and prognosis [139]. Expression profiles of inflammasomes in glioma support the involvement of NLRC4 in gliomas, and based on this, more specific functional studies have been subsequently conducted [140]. Recently, progress has been made in studying the function and molecular association of the NLRC4 inflammasome in glioma. The NLRC4 expression shows a significantly positive correlation with Tim-3 and Gal-9 expression. The Tim3-Gal-9 axis upregulates the expression and activation of the NLRC4 inflammasome to induce an inflammatory response in glioma [141]. Notably, inflammasomes are generally expressed in myeloid lineage cells, whereas NLRC4 inflammasome expression is observed in astrocytes and microglia of glioma, suggesting that astrocytes might mediate neuroinflammatory responses [139, 141]. The nature of the NLRC4 inflammasomes in glioma remains unclear. They remain functionally controversial and are associated with the NLRP3 inflammasome in other diseases [142–144]. Hence, NLRC4 should not only be investigated closely in glioma but also for additional molecular links and mechanisms, including non-canonical pathways and molecular signaling pathways.

#### NLRP6 inflammasome

NLRP6, which shows robust expression in gliomas, also belongs to the NLR family, similar to NLRP3 and NLRC4 [145]. Fewer reports of NLRP6 in glioma have been made compared to NLRP3 and NLRC4, but some progress has been made in recent years. In 2019, a clear structure of NLRP6 was elucidated using cryo-electron microscopy (cryo-EM) and crystallography, and the molecular mechanism underlying the assembly and activation of NLRP6 was elucidated, along with functional studies being undertaken in gliomas [146]. In terms of the function of the NLRP6 inflammasome, its function and associated molecules were slightly different depending on the organ in which it is expressed [147]. Importantly, the functions of the NLRP6 inflammasome in gliomas lead to a rather aggressive acceleration of carcinogenesis. NLRP6 transcriptionally induced by SP1 affects the subsequent increase in NLRP6 inflammasome activation and further causes immune escape from CD8^+^ T cells and radiation resistance of glioma cells [148]. In addition, the malignancy of gliomas has a positive correlation with the inflammatory response [61], and a significant decrease in the inflammatory response via the inhibition of NLRP6 through miR-331-3p was observed in microglial cell lines [149].

NLRP6 expression is normally regulated by upstream microbial and metabolic stimuli [150]. Recently, peroxisome proliferator-activated receptor γ (PPAR-γ) and its agonist rosiglitazone (as known to be metabolic regulator) came to be known as a representative positive regulator of NLRP6 expression [151, 152]. These regulators exhibited amplified expression in a mesenchymal subtype known to have a poor prognosis among glioblastomas (grade 4 glioma) and were suggested to be potential therapeutic target molecules [153]. So far, the roles of NLRP6 in glioma have been unveiled, and a rough outline of its overall landscape has been obtained; however, numerous aspects remain to be solved, such as the role in specific major cell types and exosomes, including the regulatory signaling pathway.

#### NLRP12 inflammasome

The first description of NLRP12 involved its contribution to inflammasome activation in response to *Yersinia Pestis* infection, which Vladimer revealed in 2012 [154]. This finding promoted the functional studies of NLRP12 that led to determining its function as an innate immune sensor and its role in other conditions, including bacterial infection, autoimmune diseases, and tumors [155–157]. Whether NLRP12 is an inhibitor or inducer of the inflammatory response is still controversial [158]. In glioma, the NLRP12 inflammasome is highly expressed. Differential expression profile analysis has shown its potential as a prognostic marker in glioblastoma [159]. Furthermore, inhibition of NLRP12 using siRNA in glioblastoma cell lines inhibited cell proliferation [159]. In contrast, in another study, NLRP12 was strongly activated in a glioma cell line in which the *SSFA2* gene was silenced. The genes strongly related to NLRP12 were found through subsequent ingenuity pathway analysis (IPA) based on microarray data [160]. Thus, pro-inflammatory molecules and cell cycle molecules showed a positive correlation with NLRP12, but IL-2 cytokines showed a very strong negative correlation [160]. Based on the above findings, although the function of NLRP12 as an innate immune sensor or an anti-inflammatory protein under various conditions is conflicting, direct or indirect evidence for its tumor-friendly characteristics in gliomas has been observed [161]. To fully understand the role of the NLPR12 inflammasome in glioma, the regulatory mechanism and additional functions must be investigated using various in vivo models.

#### AIM2 inflammasome

Absent in melanoma 2 (AIM2), a pyrin and HIN domain-containing (PYHIN) family member, is a representative inflammasome receptor protein that recognizes aberrant cytoplasmic double-strand DNA (dsDNA) [162, 163]. In the presence of a stimulus, AIM2 recruits the adaptor protein called ASC via interactions between PYDs [164–166]. This assembly induces the activity of caspase-1 and induces apoptosis as well as maturation and secretion of inflammatory cytokines, leading to innate immune responses against dsDNA of bacterial, viral, parasitic, and self-origins [164, 167–170]. Although there have been considerable studies on the pathological function and mechanism of the AIM2 inflammasome in other tumors, reports in gliomas have been relatively unclear [171]. Some functional advances have been made from the viewpoint of CNS that the AIM2 inflammasome induced by macrophages or endothelial cells in the brain pathologically leads to brain injury and chronic post-stroke cognitive impairment [172]. Notably, since 2019, the existence and function of the AIM2 inflammasome in glioma has been elucidated. AIM2 is extensively expressed in G2, G3, and G4 gliomas in TCGA database, networks with each inflammasome receptor, and epigenetic alteration patterns [140]. From functional aspects, inhibition of the AIM2 inflammasome increases the proliferation of gliomas and increases temozolomide resistance in vitro, a somewhat controversial function compared to its function in other diseases [173]. Another controversial function of AIM2 inflammasome in glioma was hinted at in the late phase of experimental autoimmune encephalomyelitis (EAE) and a mouse model of multiple sclerosis [174]. Indeed, activation of the AIM2 inflammasome in astrocytes during EAE did not alter the gene expression of apoptosis components and pro-inflammatory cytokines, suggesting distinct functional aspects for AIM2 in the CNS [174]. Meanwhile, alterations in the AIM2 inflammasome during tumor treating fields (TTFields) therapy, a non-invasive regional anti-mitotic treatment modality with minimal systemic toxicity, for glioblastoma were reported in a recent study [175]. The TTFields therapy induces AIM2 formation and activation in GSCs, leading to membrane-damaged cell death of GSCs [175]. These advances help us understand the role of the AIM2 inflammasome and suggest a direction for further study; however, it is necessary to understand the function and molecular connection of the AIM2 inflammasome with glioma in more detail and evaluate its potential as a diagnostic, prognostic, and therapeutic target molecule.

#### Other inflammasomes and glioma

In addition to the previously mentioned inflammasomes, other inflammasome complexes in gliomas have been reported. The NLRP1 inflammasome is known to be positively associated with glioma in LGG and GBM, as demonstrated by in silico analysis. In terms of the function of the NLPR1 inflammasome in CNS disease, including glioma, the NLRP1 inflammasome in the hippocampus is positively correlated with neuroinflammation and neurofibrillary formation in Alzheimer’s disease (AD) [176]. Furthermore, NLRP1 expression could affect immune cell infiltration in glioma, as observed through TIMER database analysis [177]. Interestingly, in glioma or melanoma, TMZ-induced upregulation of NLRP1 and IL-1β is linked to the Notch1 signaling pathway and, subsequently, to the acquisition of drug resistance, as revealed by MAPK inhibitors [178–182]. Some studies have focused on the presence and function of NLRP2 and NLRP7 in other tumors, but their presence in glioma is rare [183, 184]. In 2022, mutation profiling of these genes, including pyroptosis-associated genes, revealed the significant co-occurrences of mutations in glioma [185]. In particular, the NLRP2 inflammasome is expressed upon the stimulation of the damage-associated molecular pattern (DAMP) ATP in human astrocytes and is expected to play a potential role in glioma as it induces pro-inflammatory cytokine activation through NLRP2 inflammasome activation by P2X7 receptor and pannexin 1 channel [186]. In addition, studies have focused on several inflammasomes, including hypomethylation of IFI16 in glioma [187]. Although biological structures and functions of inflammasomes have been conserved according to their various origins, the investigation of their identity in gliomas should be supplemented by further study.

#### Potential therapeutic strategies for inflammasomes in glioma

In a voluminous effort, promising therapeutic results in glioma have been shown in studies targeting inflammasome. Current research focuses on inhibiting inflammasome-associated proteins involved in the priming, assembly, and activation of the inflammasome. In this section, we discuss potential therapeutic strategies targeting the inflammasome-associated molecules involved in each step of assembly and activation in glioma.

#### Targeting the priming pathway of inflammasomes

The TLR family of proteins is the most upstream receptor for the priming, assembly, and activation of the canonical or non-canonical inflammasome pathways. Previous studies have reported that TLR4 is overexpressed in astrocytes, glioma cell lines, GBM tissues, and CD133^+^ cancer stem cells [188–190]. Inhibition of TLR4 signaling with shRNA induces chemotherapy-mediated apoptosis of glioma CD133^+^ cancer stem cells [191]. p65 nuclear translocation by non-canonical TLR4 signal/activation of DNA repair genes is positively correlated with the survival of U87MG glioma cells, suggesting that p65 is a potential therapeutic target for the inflammasome [192]. TLR2 also plays pathological roles in glioma, including immune evasion and the development and progression of glioma cells [99, 193]. Treatments targeting the expression or activation of TLR2 in GSCs within the glioma may be efficient strategies [107]. TRIF and MyD88, which are intracellular adaptor proteins of TLR, can also be targeted with inflammasome therapy in glioma, leading to the destruction of the TLR and NF-κB loop to sustain an inflammatory response [194]. Aberrant activation of NF-κB, a downstream transcription factor of the TLR pathway, is a hallmark of glioma [195]. Inhibition of these signaling pathways results in significant programmed death of glioma cells, and these inhibitors can be used as therapeutic adjuvants to the TMZ standard chemotherapy for glioma [196]. Inhibitors of TLR and NF-κB, the key molecules in the inflammasome priming step, have been described earlier, but their efficacy in gliomas and the mode of action in the inflammasome axis are still unclear [197, 198]. In addition, indirect inflammasome inhibition by targeting multiple elements that regulate the priming step, including ROS, hypoxia, metabolites, lipid metabolites, and complement proteins, although not well known in glioma, can be expected with novel therapeutic approaches [199]. For the clinical application of candidate drugs targeting these priming step-related molecules, additional in vitro and in vivo validations are essential.

#### Targeting the assembly and activation of the inflammasomes

A promising therapeutic strategy targeting receptor proteins that play an important role in assembling and activating inflammasomes was suggested earlier [200]. Representatively, numerous pharmacological inhibitors of the NLRP3 inflammasome have been described [201]. Indirect or direct inhibitors of NLRP3 involve Glyburide, JC124, FC11A-2, Parthenolide, VX765, BHB, MCC950, and Tranilast [201]. Although candidate agents targeting receptor proteins in gliomas are relatively unclear compared to such agents in other diseases, some assessments have been performed. BHB, WP1066, and IP-Se-06 were found to directly inhibit glioma migration, proliferation, and viability by inhibiting the expression or activity of the NLRP3 inflammasome in glioma [127–129]. Additionally, miR-331-3p, SP1 inhibitor, and PPAR-γ inhibitors can prevent the expression of NLRP6 in glioma [131, 148, 149]. Inhibition of P2X7 and pannexin 1 also reduces the inflammatory response of gliomas by inhibiting the expression of the NLRP2 inflammasome [186, 202].

In a recent study, inhibition of TIM-3, an immune checkpoint molecule robustly expressed in glioma, was suggested as a therapeutic strategy to inhibit the NLRC4 inflammasome in glioma cells [139, 203]. In fact, in silico and in vitro validation results regarding the Tim-3/Gal-9 axis and the NLRC4 inflammasome showed a significantly positive correlation according to the WHO glioma grade, and it was found that Tim-3 regulates the expression and activity of the NLRC4 inflammasome [141]. These results provide potential insights into the networks between various biological mechanisms and inflammasomes and implicate dual-acting therapeutic strategies involving target therapy for inflammasomes and other previously known mechanisms.

Notably, there is evidence suggesting that adaptor proteins of the inflammasome are potential therapeutic targets for glioma [204]. For example, PYCARD, known as apoptosis-associated speck-like protein containing a CARD (ASC), plays a role as an adaptor to bridge sensor proteins and effector molecules [88]. A CARD-associated risk score (CARS) was positively correlated with the poor prognosis of glioma patients who underwent standard therapy [205]. These advances using an inflammasome-associated gene set allow predicting the therapeutic potency in glioma patients.

The role of berberine, a potential therapeutic agent targeting caspase-1, an important hall marker of inflammasome activity, was investigated in a glioma cell line [114]. Berberine directly inhibits caspase-1 activation via the ERK1/2 signaling pathway in glioma cells, leading to inhibition of the expression of pro-inflammatory cytokines such as IL-1β and IL-18 [114]. Anakinra, a recombinant IL-1 receptor agonist, is a representative drug targeting aggressive inflammation [206]. Anakinra inhibited the expression of IL-1β in tumor cells and PBMCs of GBM, inhibited the proliferation and migration of tumor cells, and reduced inflammatory signals [207].

#### Therapeutic aspects for the non-canonical inflammasome pathway

Although fewer inhibitors of the non-canonical inflammasome-pathway-associated molecules have been identified compared with those for canonical inflammasome-pathway-associated molecules, they exhibit biological evidence as potential prognostic biomarkers [208]. The transcriptional level of GSDMD in gliomas increased according to the WHO grade, and it was verified as a prognostic marker through survival analysis, Cox-regression analysis, and histological staining [208]. Importantly, the expression pattern of GSDMD showed differences according to the status of IDH1/2 mutation and 1p19q co-deletion, indicating detailed molecular characteristics of gliomas and the biological link of the inflammasome [208]. Based on these advances, the potential characteristics of the inflammasome according to the molecular and histological subtype of glioma can serve as a promising therapeutic candidate target for personalized therapy. One limitation in the research on the non-canonical pathway is that caspase-4 and caspase-5, the key regulators of the non-canonical inflammasome, have not yet been explored in gliomas; thus, functional studies on these are urgently required. Collectively, we summarized the potential therapeutic candidates for target molecules in the inflammasome axis (Table 1).

**Table 1:** Potential therapeutic candidates for inflammasome-associated molecules in glioma

Molecule	Targets	Mechanism of action	Reference
OPN-305	TLR2	inhibit TLR2 mediated proinflammatory response	[209]
T2.5	TLR2	inhibit TLR2 mediated proinflammatory response	[210]
Candesartan	TLR2/4	suppress Pam3CSK4 and LPS induced TLR2/4 activation	[211]
TAK-242/Resatorvid	TLR4	inhibit TLR4 signaling pathway	[212]
Valsartan	TLR4	inhibit TLR4 signaling pathway	[213]
Simvastatin	TLR4	inhibit TLR4 activity	[214]
Atorvastatin	TLR4	inhibit TLR4 activity	[215]
NI-0101	TLR4	block TLR4 dimerization	[216]
ST2825	MyD88	inhibit MyD88 dimerization	[217]
Parthenolide	NF-κB	downregulate the phosphorylation of NF-κB	[218]
Andrographolide	NF-κB	inhibit TLR/NF-κB signaling pathway	[219]
P2X7R antagonist	P2X7R	block the P2X7R	[220]
DPP9	NLRP1	block the UPA-mediated formation of functional UPA-CARD filament	[221]
Oxysterol	NLRP2	impair the activation of NLRP2	[222]
Glyburide	NLRP3	inhibit NLRP3 inflammasome activation	[223]
16,673–34-0	NLRP3	inhibit NLRP3 inflammasome formation	[201]
JC124	NLRP3	reduce NLRP3 expression	[224]
Bay 11–7082	NLRP3	prevent NLRP3 inflammasome organization	[225]
BHB	NLRP3	inhibit NLRP3 activity	[127]
WP1066	NLRP3	inhibit NLRP3 activity	[128]
IP-Se-06	NLRP3	inhibit NLRP3 activity	[129]
MCC950	NLRP3	block both canonical and non-canonical NLRP3 inflammasome activation	[226]
MNS	NLRP3	suppress ATPase activity of NLRP3	[227]
CY-09	NLRP3	block NLRP3 inflammasome activation	[227]
Tranilast	NLRP3	impair endogenous NLRP3-ASC interaction	[227]
OLT1177	NLRP3	inhibit both canonical and non-canonical NLRP3 inflammasome activation	[228]
Oridonin	NLRP3	inhibit NLRP3 inflammasome activation	[229]
β -hydroxybutyrate	NLRP3	inhibit NLRP3 activation by disrupting NLRP3-ASC oligomerization	[230]
Genipin	NLRP3 aNLRC4	inhibit NLRP3 and NLRC4 inflammasome activation via autophagy suppression	[231]
BI8622 (HUWE1 inhibitor)	NLRP3 NLRC4 AIM2	inhibit inflammasome activation	[232]
C75, C77, C78	NLRC4	inhibit NLRC4 activity	[233]
mir-331-3p	NLRP6	inhibit NLRP6 expression	[149]
ROS (AIP1 mediated)	NLRP6	inhibit NLRP6 expression	[234]
CRH	NLRP6	inhibit NLRP6 expression	[235]
mir-372	NLRP12	inhibit NLRP12 expression	[236]
IFI16-β	AIM2	inhibit dsDNA-induced AIM2 inflammasome activation	[237]
CRID3	AIM2	prevent AIM2-dependent pyroptosis	[220]
STING	IFI16	ubiquitination and degradation of IFI16	[238]
Gö6976 (pUL97 inhibitor)	IFI16	suppress IFI16 re-localization	[239]
MM01	ASC	interferes with ASC speck formation	[240]
FC11A-2	caspase-1	block the proximity-induced autocleavage of procaspase-1	[225]
Berberine	caspase-1	inhibit caspase-1 activation	[114]
Thalidomide	caspase-1	inhibit caspase-1 activation	[241]
VX740/Pralnacasan	caspase-1	inhibit caspase-1 activation	[242]
VX765/Belnacasan	caspase-1	inhibit caspase-1 activation	[243]
Ac-YVAD-CHO	caspase-1	inhibit caspase-1 activation	[244]
Z-LEVD-FMK	caspase-4	inhibit caspase-4 expression	[245]
Z-WEHD-FMK	caspase-5	inhibit caspase-5 activation	[246]
Disulfiram	GSDMD	inhibit GSDMD cleavage and GSDMD-mediated pore formation	[247]
Necrosulfonamide	GSDMD	inhibit GSDMD-mediated pore formation	[248]
Canakinumab	IL-1β	block the biologic activity of IL-1β	[241]
Rilonacept	IL-1β	block the biologic activity of IL-1β	[249]
Gevokizumab	IL-1β	block the biologic activity of IL-1β	[250]
LY2189102	IL-1β	block the biologic activity of IL-1β	[251]
Anakinra	IL-1R and IL-18	block the biologic activity of IL-1β and IL-18	[252]

## Conclusions

The biological findings on how inflammasomes are activated in tumors have increased their clinical importance. Importantly, an understanding of the balance between beneficial and detrimental inflammasome in tumor cells is essential. In particular, inflammasome activity reinforces tumor progression and invasion in glioma. However, activation of not all inflammasome proteins can be considered harmful in glioma, and the therapeutic inhibition of this axis has to be balanced against its beneficial contribution.

Previous studies on the role of inflammasomes in glioma have provided fragmentary approaches and have not yet led to any clinical significance. However, further mechanistic insights into the role of the inflammasome in glioma will provide opportunities to develop therapies for patients with other inflammatory CNS diseases. In addition, clarification of the association between the inflammasome and its underlying mechanisms in glioma may indicate a new direction for glioma diagnosis, prognosis, and therapy.

## Data Availability

Not applicable.

## Abbreviations

AD: Alzheimer’s Disease
AIM2: Absent in Melanoma 2
ALD: Alendronate
APC: Astrocyte Precursor Cell
ASC: Apoptosis-associated Speck-like protein containing a CARD
ATRX: Alpha-Thalassemia/Mental Retardation, X-linked
BBB: Blood–Brain Barrier
bFGF: Basic Fibroblast Growth Factor
BHB: Beta-Hydroxybutyrate
BRB: Blood-Retinal Barrier
CCL12: C–C Motif Chemokine Ligand 12
CCL2: C–C Motif Chemokine Ligand 2
CCL3: C–C Motif Chemokine Ligand 3
CCL4: C–C Motif Chemokine Ligand 4
CCL5: C–C Motif Chemokine Ligand 5
CGGA: Chinese Glioma Genome Atlas
CIC: Capicua Transcriptional Repressor
CNS: Central Nervous System
cryo-EM: Cryo-Electron Microscopy
CXCL10: C-X-C Motif Chemokine Ligand 10
DAMP: Damage Associated Molecular Pattern
EAE: Experimental Autoimmune Encephalomyeliti
EGFR: Epidermal Growth Factor Receptor
ERK: Extracellular signal-regulated Kinases
EV-71: Enterovirus 71
Gal-9: Galectin-9
GAMs: Glioma Associated Microglia/Macrophages
GRPC: Glial Restricted Progenitor Cell
GSC: Glioma Stem Cell
HIF-α: Hypoxia-Inducible Factor-1
H-RAS: Harvey Rat Sarcoma Virus
IDH1/2: Isocitrate Dehydrogenase ½
IFI16: Interferon Gamma Inducible Protein 16
IL-12: Interleukin 12
IL-18: Interleukin 18; IL-1β, Interleukin 1 beta
IL-2: Interleukin 2
IL-23: Interleukin 23
IL-6: Interleukin 6
IPA: Ingenuity Pathway Analysis
JAK: Janus Activated Kinase
LGG: Low Grade Glioma
MAPK: Mitogen‑Activated Protein Kinase
MDSC: Myeloid-Derived Suppressor Cells
MGMT: O-6-Methylguanine-DNA Methyltransferase
MHC: Major Histocompatibility Complex
MS: Multiple Sclerosis
NF-κB: Nuclear Factor-κB
NG2: Neuron-glial Antigen 2
NLR: Nucleotide-binding and Leucine-rich Repeat
NLRC4: NLR Family CARD Domain Containing 4
NLRP3: NLR Family Pyrin Domain Containing Protein 3
NSC: Neural Stem Cell
OLIG2: Oligodendrocyte Transcription Factor 2
OPC: Oligodendrocyte Precursor Cells
pDC: Plasmacytoid Dendritic Cell
PDGF-AA: Platelet-Derived Growth Factor AA
PDGFRα: Platelet-Derived Growth Factor Receptor A
PNS: Peripheral Nervous System
PPAR-γ: Peroxisome Proliferator-Activated Receptor γ
PRR: Pattern Recognition Receptors
PTEN: Phosphatase and Tension Homolog
PTM: Post-Translational Modification
PYD: Pyrin Domain
PYHIN: Pyrin and HIN domain-containing
RAS: Rat Sarcoma Virus
RLR: RIG-I-like Receptor
ROS: Reactive Oxygen Species
SP1: Specificity Protein 1
SSFA2: Sperm-Specific Antigen 2
STAT3: Signal Transducer and Activator of Transcription 3
T3SS: Type III Secretion System
TCGA: The Cancer Genome Atlas
TERT: Telomerase Reverse Transcriptase
TGF- β: Transforming Growth Factor-beta
Tim-3: T cell Immunoglobulin and Mucin-domain containing-3
TIMER: Tumor Immune Estimation Resource
TLR: Toll-like Receptor
TME: Tumor Microenvironment
TNF-α: Tumor Necrosis Factor-α
TP53: Tumor Protein p53
TTFields: Tumor Treating Fields
VEGF: Vascular Endothelial Growth Factor
WHO: World Health Organization
